# The Mutation of *piezo1* Weakens the Intermuscular Bones in Zebrafish and Crucian Carp

**DOI:** 10.3390/ijms262210851

**Published:** 2025-11-08

**Authors:** Xinyu Zhang, Jinyuan Che, Zhuang Li, Baolong Bao, Chunxin Fan

**Affiliations:** 1 Key Laboratory of Exploration and Utilization of Aquatic Genetic Resources, Ministry of Education, Shanghai Ocean University, Shanghai 201306, China; 2 International Research Center for Marine Biosciences, Ministry of Science and Technology, Shanghai Ocean University, Shanghai 201306, China; 3 National Demonstration Center for Experimental Fisheries Science Education, Shanghai Ocean University, Shanghai 201306, China

**Keywords:** intermuscular bones, CRISPR/Cas9, mechanosensitive ion channels, zebrafish, crucian carp

## Abstract

Intermuscular bones (IBs), unique skeletal features found only in teleost fishes, pose significant challenges to food processing and consumption. While recent studies have identified several key genetic regulators of IB development, the role of mechanosensory mechanisms remains largely unexplored. This study investigated the role of Piezo1, a critical mechanosensitive ion channel, in IB formation using zebrafish and crucian carp models. Our findings demonstrated that *piezo1* was expressed in the myoseptum of zebrafish, and CRISPR/Cas9-mediated knockout of this gene resulted in shorter and smaller IBs. Similar knockout experiments in crucian carp confirmed the conserved role of Piezo1 across cyprinid species. These results established Piezo1 as a key regulator of IB development, providing new insights into the molecular mechanisms underlying this process and suggesting potential strategies for breeding IB-free fish strains through modulation of mechanosensory pathways.

## 1. Introduction

Intermuscular bones (IBs), also known as intermuscular spiny bones, are unique bony structures found exclusively in teleost fishes. These bones develop through ossification of the myoseptal connective tissue located adjacent to the vertebral column [1,2,3]. Based on their anatomical positions, IBs are classified into epineurals, epipleurals, and epicentrals [2,4]. The distribution and morphology of IBs vary remarkably across teleost lineages, displaying strong phylogenetic patterns. Lower teleosts, such as species within the *Clupeiformes* and *Cypriniformes*, typically possess numerous, well-developed IBs, whereas higher teleosts, including *Perciformes* and *Tetraodontiformes*, exhibit drastically reduced or entirely absent IBs [3,5].

IBs complicate fish processing, increase the risk of choking during consumption, and reduce consumer acceptance of fish products. These practical concerns have motivated intensive research into the developmental mechanisms underlying IB formation, with the goal of reducing or eliminating IBs in farmed species through selective breeding or biotechnological approaches [6,7]. Recent advances in gene-editing technologies, particularly CRISPR/Cas9, have greatly facilitated the identification of genetic regulators involved in IB development. Targeted mutagenesis has been employed in key osteogenic genes (e.g., *bmp6*, *scxa*, *runx2b*) to reduce or eliminate IBs in commercially important cyprinids, such as crucian carp, grass carp, blunt snout bream, and silver carp [8,9,10,11,12,13,14,15].

Beyond intrinsic genetic programs, skeletal development is strongly modulated by mechanical stimuli, as exemplified by the bone-strengthening effects of physical activity [16,17,18]. A close relationship exists between swimming mechanics and IB ontogeny: in anguilliform (eel-like) swimmers, IB ossification begins anteriorly, whereas in carangiform (mackerel-like) swimmers, it initiates posteriorly. Experimental disruption of normal swimming, such as tail resection in zebrafish, leads to a pronounced reduction in IB ossification [19]. These findings suggest that mechanical signaling plays a critical role in IB formation.

The Piezo family of mechanosensitive ion channels, comprising Piezo1 and Piezo2, serves as a key mediator of mechanical transduction [20]. *piezo1* is mainly expressed in non-sensory tissues, including vascular system and mesenchyme [21,22,23], and regulates the cell fate of mesenchymal stem cells [22]. In mammals, Piezo1 is essential for bone homeostasis; Piezo1-deficient mice display osteopenia and increased bone fragility, underscoring its importance in skeleton development [23,24,25]. In addition, double knockout of *piezo1* and *piezo2a* leads to congenital systemic malformations in zebrafish [26]. However, whether Piezo1 participates in the formation of teleost-specific IBs remains unknown.

In this study, we detected *piezo1* expression in the myoseptum by in situ hybridization using zebrafish as a model. We knocked out *piezo1* and *piezo2b* in zebrafish using CRISPR/Cas9 and found that the mutation of *piezo1* resulted in shorter and smaller IBs. To test the evolutionary conservation of this mechanism, we knocked out *piezo1* homologs using crispant in crucian carp and observed similar attenuation of IB formation. Together, these results identify Piezo1 as a crucial regulator of IB development, providing new mechanistic insights into the genetic and biomechanical control of this teleost-specific skeletal structure and offering a promising molecular target for the breeding of IB-free fish.

## 2. Results

### 2.1. piezo1 Is Expressed in Zebrafish Tendons

**Figure 1:**
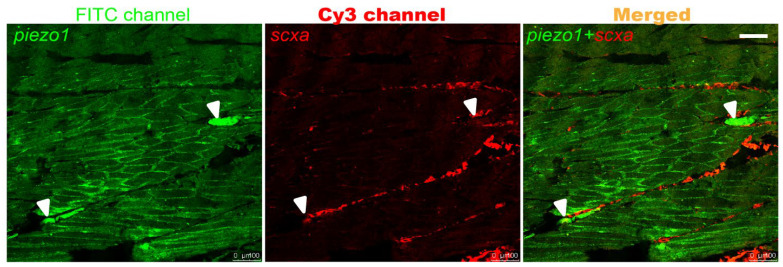
FISH on adult zebrafish caudal peduncle sections. White arrows indicate the expression sites of *piezo1*. Scale bar: 100 μm.

To investigate the expression pattern of *piezo1*, paraffin section fluorescence in situ hybridization (FISH) was performed on the caudal peduncle of adult zebrafish. FISH analysis revealed that the *piezo1* signal co-localized with *scxa* signals in the tendons and exhibited a punctate pattern (Figure 1). This pattern corresponded to the expected location of IBs. This suggests that *piezo1* plays a role in IB development.

### 2.2. piezo1 Mutation Significantly Decreases the Area and Length of IBs in Zebrafish

**Figure 2:**
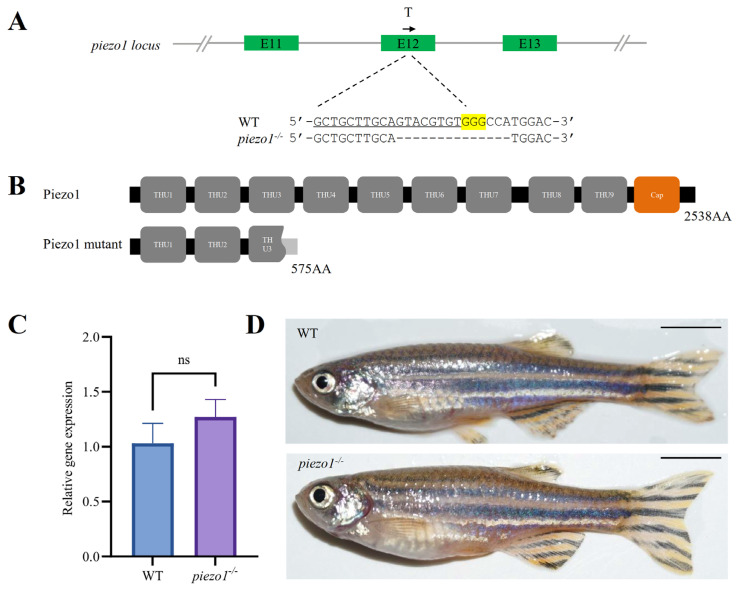
Generation of *piezo1* homozygous mutant zebrafish. (**A**) Schematic of the guide RNA (gRNA) target site and the resulting mutant sequence in zebrafish *piezo1*. T indicates the position of gRNA target, and the black arrow represents the direction of gRNA target. The single guide RNAs (sgRNA) sequence is underlined, the PAM is highlighted in yellow, and the deleted bases are represented by dashes. (**B**) Domain architecture of the predicted Piezo1 protein in WT and mutants. (**C**) qRT-PCR analysis of *piezo1* gene expression in *piezo1* mutant and WT control larvae at 3 days post-fertilization (dpf). ns, not significant. (**D**) Lateral view of adult WT and *piezo1* homozygous mutant zebrafish. Scale bar: 5 mm.

We used the CRISPR/Cas9 system to knockout *piezo1* gene in zebrafish. Based on the confirmed targeting efficiency by fluorescence PCR-coupled capillary electrophoresis (Supplementary Figure S1A), we established a homozygous mutant line carrying a 14-base pair (bp) deletion in exon 12 (Figure 2A). This deletion led to a premature termination codon, producing a truncated protein of 575 amino acids (Figure 2B). Quantitative Real-Time PCR (qRT-PCR) revealed no significant difference in *piezo1* mRNA levels between homozygous mutants and wild-type (WT) controls, suggesting that the nonsense-mediated mRNA decay was not induced (Figure 2C). Additionally, the *piezo1* homozygous mutants exhibited no significant differences in overall body morphology compared to WT zebrafish (Figure 2D).

**Figure 3:**
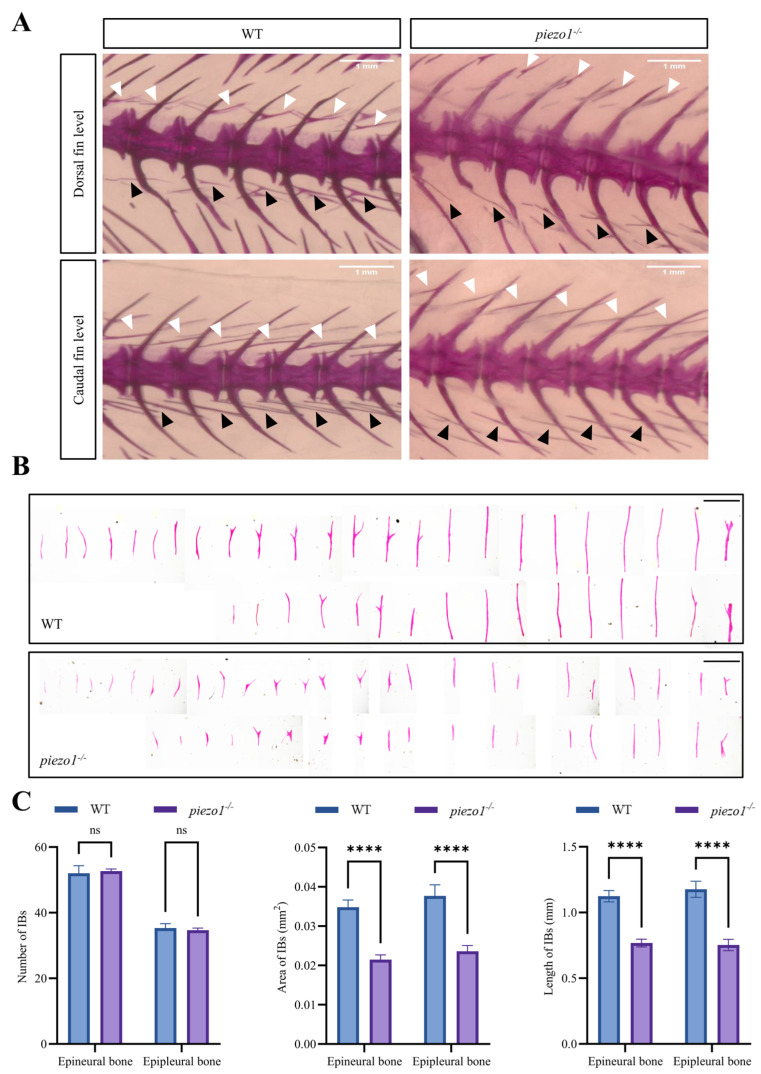
Alizarin red S staining of *piezo1* homozygous mutant and WT zebrafish. (**A**) Representative images of IBs surrounding the dorsal and caudal fins in *piezo1* homozygous mutants and WT controls. White arrows indicate epineural bones, black arrows indicate epipleural bones. Scale bar: 1 mm. (**B**) Isolated IBs from *piezo1* homozygous mutants and WT controls. Scale bar: 1 mm. (**C**) Quantification of the number, area, and length of isolated IBs. ****, *p* < 0.0001; ns, not significant. *n* = 3.

Alizarin Red S staining was used to visualize IBs in *piezo1* homozygous mutant zebrafish (Figure 3A,B). Quantitative analyses showed significant reductions in the area (epineural bone: WT, 0.035 ± 0.016 mm^2^ vs. *piezo1^−/−^*, 0.021 ± 0.02 mm^2^; epipleural bone: WT, 0.037 ± 0.018 mm^2^ vs. *piezo1^−/−^*, 0.023 ± 0.015 mm^2^) and length (epineural bone: WT, 1.124 ± 0.56 mm vs. *piezo1^−/−^*, 0.767 ± 0.543 mm; epipleural bone: WT, 1.177 ± 0.688 mm vs. *piezo1^−/−^*, 0.75 ± 0.605 mm), but not the number (epineural bone: WT, 52 ± 4 vs. *piezo1^−/−^*, 52.67 ± 1.33; epipleural bone: WT, 35.3 ± 2.7 vs. *piezo1^−/−^*, 34.67 ± 1.33), of IBs in *piezo1* mutants compared to WT controls (Figure 3C).

### 2.3. piezo2b Mutation Does Not Affect IB Development in Zebrafish

**Figure 4:**
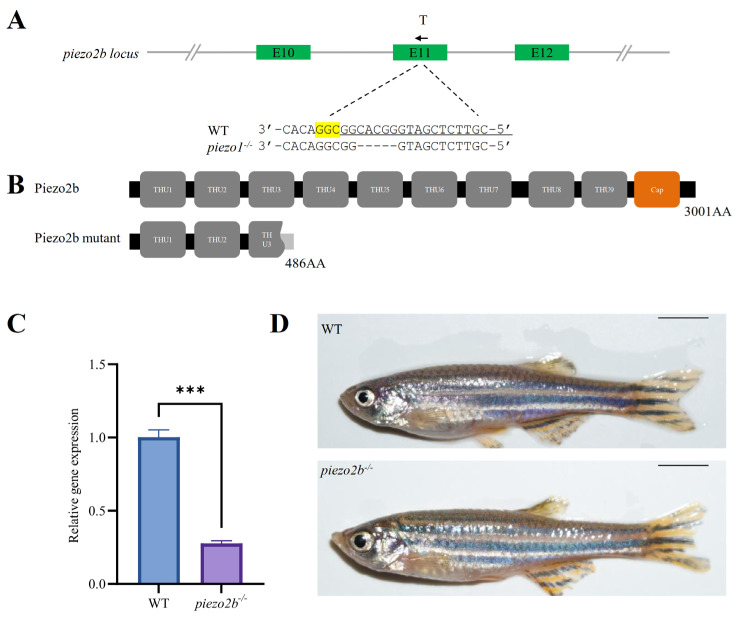
Generation of *piezo2b* homozygous mutant zebrafish. (**A**) Schematic of the gRNA target site and the resulting mutant sequence in *piezo2b* locus. T indicates the position of gRNA target, and the black arrow represents the direction of gRNA target. The sgRNA targeting sequence is underlined, the PAM sequence is highlighted in yellow, and dashes indicate the deletion nucleotides. (**B**) Domain architecture of the predicted Piezo2b in WT and homozygous mutant. (**C**) qRT-PCR analysis of *piezo2b* gene expression in *piezo2b* mutants and WT control larvae at 3 dpf. ***, *p* < 0.001. (**D**) Lateral view of adult WT and *piezo2b* homozygous mutants. Scale bar: 5 mm.

**Figure 5:**
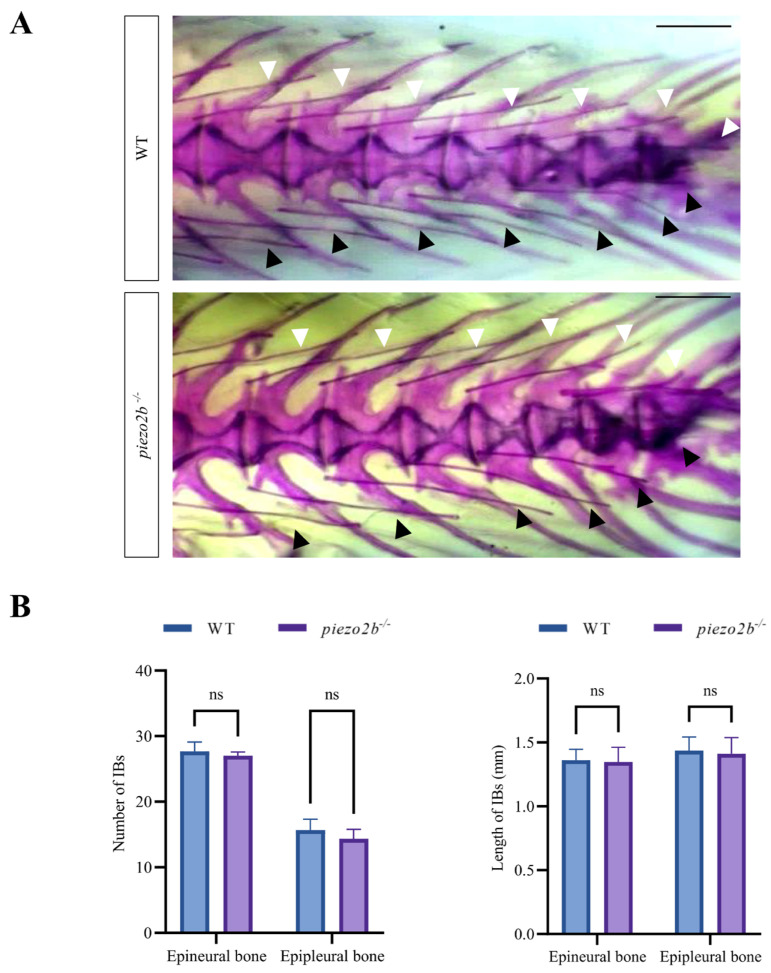
Alizarin Red S staining of *piezo2b* homozygous mutants and WT zebrafish. (**A**) Representative images IBs in the tail regions between *piezo2b* homozygous mutants and WT individuals. White arrows indicate epineural bones, black arrows indicate epipleural bones. Scale bar: 1 mm. (**B**) Quantitative analysis of the number and length of IBs in *piezo2b* mutants and WT controls. ns, not significant. *n* = 3.

We disrupted the *piezo2b* gene in zebrafish using CRISPR/Cas9. The targeting efficiency was first confirmed by fluorescent PCR-coupled capillary electrophoresis (Supplementary Figure S1B), and then a homozygous mutant line carrying a 5-bp deletion in exon 11 was generated (Figure 4A). The deletion introduced a premature termination codon, resulting in a truncated Piezo2b protein of 486 amino acids (Figure 4B). qRT-PCR analysis revealed a significant reduction in *piezo2b* mRNA levels in *piezo2b* homozygous mutants compared to WT controls, indicating that the mutation induces nonsense-mediated mRNA decay (Figure 4C). Compared to WT individuals, *piezo2b* mutants showed no obvious changes in body size (Figure 4D). The development of IBs was also not changed significantly in *piezo2b* mutants as assessed by Alizarin Red S staining (Figure 5A,B).

### 2.4. piezo1 Homologs Knockout Using Crispant in Crucian Carp

**Figure 6:**
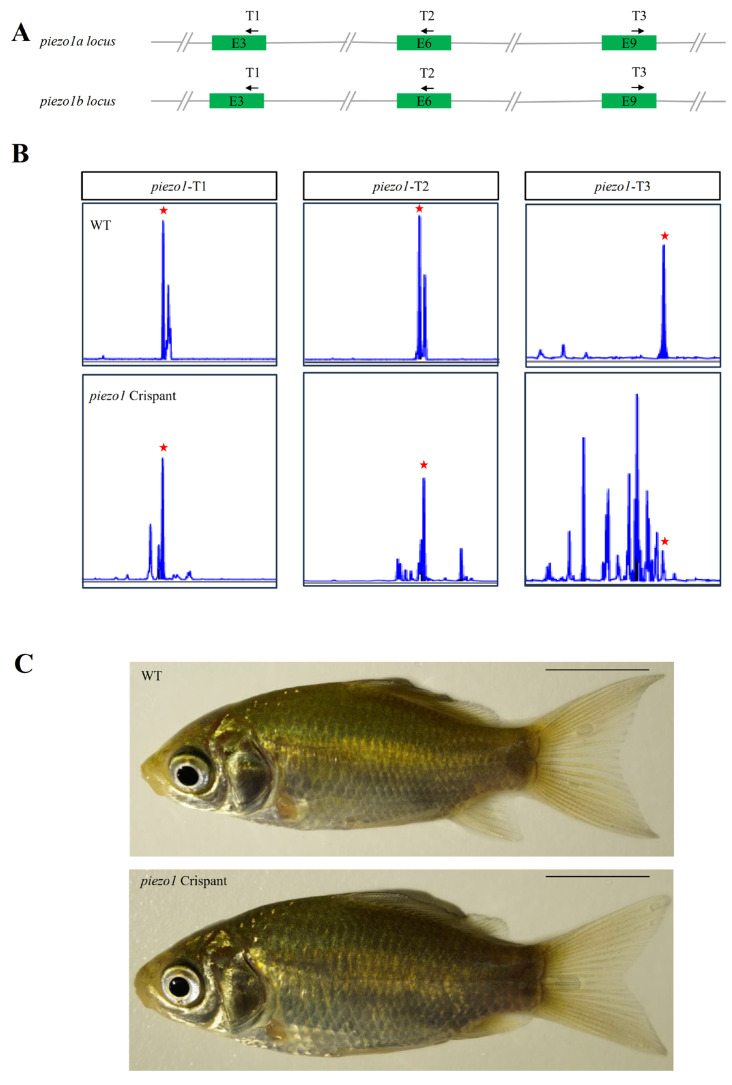
Generation of *piezo1* mutant crucian carp. (**A**) Schematic of the crispant targeting strategy for three gRNA target sites in *piezo1* gene loci in crucian carp. T1, T2 and T3 indicate the position of gRNA targets, and the black arrows represent the direction of gRNA targets. (**B**) Capillary electrophoresis genotyping of the three gRNA target sites. The x-axes (size) and y-axes (signal intensity) of the peak diagrams for WT and mutant fish are aligned. Red stars indicate the position of the WT peak. (**C**) Lateral view of WT and mutant crucian carp at 3 months post-fertilization. Scale bar: 1 cm.

To rapidly assess the function of *piezo1* in crucian carp, we generated crispant fish by co-injecting Cas9 protein and three sgRNAs targeting the two *piezo1* homologs: *piezo1a* (ENSYGUG00000015181) and *piezo1b* (ENSYGUG00000047551) (Figure 6A). To assess editing efficiency, we analyzed the target regions by fluorescence PCR-coupled capillary electrophoresis, which revealed a spectrum of deletions and insertions at all three target sites, confirming successful *piezo1* knockout in crucian carp (Figure 6B). Nevertheless, *piezo1* crispant crucian carp exhibited normal body size compared to WT controls (Figure 6C).

**Figure 7:**
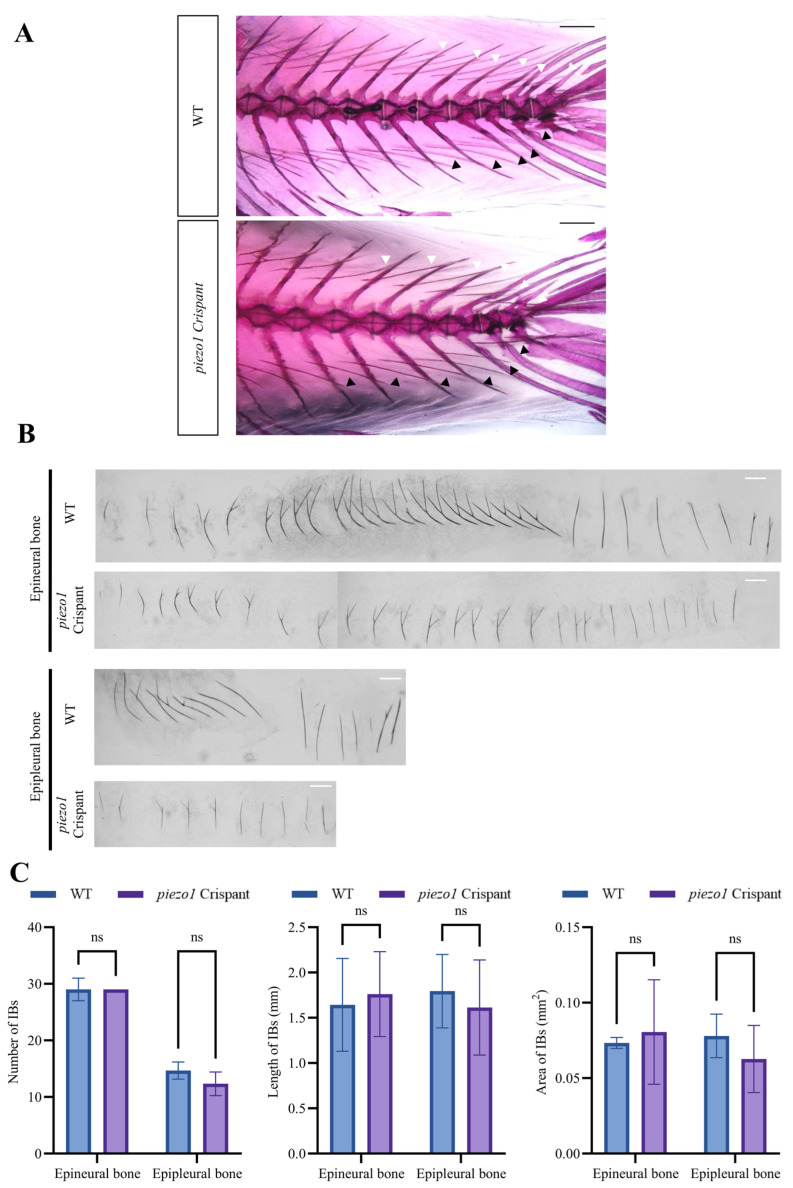
Analysis of IB phenotype in *piezo1* crispants and WT crucian carps. (**A**) Representative images of Alizarin red S staining showing IBs in *piezo1* crispants and WT individuals. White arrows indicate epineural bones, black arrows indicate epipleural bones. Scale bar: 1 mm. (**B**) Representative images of isolated IBs of a *piezo1* crispant and a wild-type crucian carp. Scale bar: 1 mm. (**C**) Quantitative analysis of IB number, length, and area in *piezo1* crispants and WT crucian carps. ns, not significant. *n* = 3.

Alizarin Red S staining revealed that some individual mutants exhibited a reduction in IB size (Figure 7A,B). *piezo1* crispants exhibited a decreasing trend in the number (epineural bone: WT, 29 ± 2 vs. *piezo1* crispants, 29; epipleural bone: WT, 14.67 ± 1.33 vs. *piezo1* crispants, 12.33 ± 1.67), length (epineural bone: WT, 1.642 ± 0.488 mm vs. *piezo1* crispants, 1.761 ± 0.99 mm; epipleural bone: WT, 1.792 ± 0.598 mm vs. *piezo1* crispants, 1.613 ± 1.117 mm), and area (epineural bone: WT, 0.074 ± 0.076 mm^2^ vs. *piezo1* crispants, 0.08 ± 0.103 mm^2^; epipleural bone: WT, 0.078 ± 0.063 mm^2^ vs. *piezo1* crispants, 0.0627 ± 0.05 mm^2^) of IBs compared to the WT controls. Despite the lack of statistical significance, this trend nonetheless suggests that Piezo1 may play a subtle role in intermuscular bone formation (Figure 7C).

## 3. Discussion

IBs represent a unique skeletal structure in teleosts, originating from the connective tissue of the myosepta. While significant progress has been made in identifying genetic regulators such as *bmp6* and *runx2b* that are crucial for IB development [8,13], these genes are highly conserved and ubiquitous across teleosts and other vertebrates. This high degree of conservation suggests that sequence variations in these core osteogenic genes are unlikely to be the primary drivers behind the evolutionary trajectory of IBs. Danos and Ward, through a broad comparative analysis of teleost characteristics, proposed a potential link between biomechanical forces and IB morphology [16]. Supporting this, Yao et al. (2015) provided empirical evidence by demonstrating that the ossification pattern of IBs differs significantly between fish with distinct swimming modes (anguilliform vs. carangiform), directly implicating mechanical load in their development [19]. Our findings provide direct functional evidence for this hypothesis. We demonstrate that the loss of the mechanosensory protein Piezo1 leads to shorter and smaller IBs in both zebrafish and crucian carp. This conserved phenotype across two cyprinid species strongly suggests that mechanosensation is a fundamental mechanism underpinning IB development. Therefore, we propose that mechanical forces, transduced through channels like Piezo1, may have played a pivotal role not only in the developmental regulation but also in the evolutionary history of IBs in teleosts.

The Piezo1 channel is a key mediator of mechanotransduction, converting mechanical stimuli into intracellular biochemical signals through conformational changes that alter membrane potential [20]. It is broadly expressed in non-sensory mesenchymal cells, including osteoblasts. Our results align with a growing body of evidence from mammalian models, where *piezo1* deletion in osteoblasts results in significant bone loss in the femur and tibia [22,23,24]. The observation that *piezo1* is expressed in IBs and its mutation leads to impaired IB development in both zebrafish and crucian carp underscores a deeply conserved role for Piezo1 in skeletal formation across vertebrates, despite the vast morphological differences between mammalian long bones and teleost intermuscular bones. Studies in mice have shown that Piezo1 promotes bone formation by activating key signaling pathways such as YAP1 and β-catenin [25]. However, the precise molecular pathway through which it exerts its effects in fish remains an open question. Therefore, a key direction for future research will be to elucidate the detailed mechanistic link between Piezo1-mediated mechanosensation and the transcriptional program governing osteoblast differentiation and mineralization during IB formation. Unraveling this mechanism will be crucial for a comprehensive understanding of how mechanical forces shape this distinctive teleost trait.

A notable finding of our study is that the *piezo1* mutation specifically affects IBs, causing them to be shorter and smaller, while other skeletal elements appear largely normal. We propose two explanations for this phenotype-specificity. First, the mechanical environment may dictate the requirement for Piezo1. The vertebral column bears less compressive loads, which may be less dependent on Piezo1-mediated mechanosensing. In contrast, IBs are embedded within the dynamic myoseptal connective tissue, which experiences significant and complex tensile and shear stresses generated during continuous muscle contraction and swimming. Consequently, IBs might be more sensitive to *piezo1* mutations. This is similar to observations in *Piezo1* mutant mice, where long bones like the femur and ulna, which bear high mechanical loads, are more severely affected than the vertebrae [23]. Second, functional redundancy or compensation by the related channel, Piezo2, could mask broader skeletal defects. Ramly et al. demonstrated that double knockout of *piezo1* and *piezo2a* in zebrafish results in systemic skeletal deformities during early development that are far more severe than either single mutant [26]. This genetic interaction strongly suggests that Piezo2 can compensate for the loss of Piezo1 in certain skeletal contexts, potentially explaining the limited phenotype in our *piezo1* single mutants. Future studies creating tissue-specific double knockouts will be essential to dissect the individual and combined contributions of these mechanosensors to the entire fish skeleton.

Zebrafish lacking functional Piezo1 exhibited significantly shorter and thinner IBs, whereas *piezo1* crispant crucian carps showed a decreasing trend in IBs, though statistically insignificant. We propose several explanations for this discrepancy. First, the CRISPR/Cas9-mediated knockout in F0-generation crucian carp (Crispants) may have exhibited incomplete mutagenesis efficiency, resulting in a mosaic presence of WT *piezo1* in a subset of cells, thereby attenuating the overall phenotypic penetrance. Second, unlike zebrafish, the *piezo1* gene in crucian carp has undergone duplication, yielding two paralogs—*piezo1a* and *piezo1b*—which might exhibit functional redundancy or differential expression. Although we designed sgRNAs to simultaneously target both genes, the efficiency of achieving high efficient knockout was compromised. Third, the current sample size may have been insufficient to detect subtle phenotypic alterations, limiting the statistical power of our observations. Further investigations employing stable mutant lines and expanded sample sizes will help clarify Piezo1’s role in intermuscular bone development in cyprinid fishes.

## 4. Materials and Methods

### 4.1. Experimental Animals

The zebrafish (*Danio rerio*) used in this study were 3-month-old individuals of the AB strain, obtained from the China Zebrafish Resource Center (Wuhan, China). All zebrafish were maintained under controlled conditions at a constant temperature of 28 °C and a photoperiod of 14 h of light followed by 10 h of darkness. Sexually mature female and male zebrafish were paired at 28 °C to obtain embryos. The embryos were incubated in a constant temperature and humidity incubator at 28.5 °C. Paramecia were fed to the larvae starting at 7 dpf, and from 14 dpf, the larvae were fed with brine shrimps and simultaneously transferred to the zebrafish rearing system.

The crucian carp (*Carassius auratus*) used were diploid individuals sourced from Shanghai Dianyuan Aquaculture Farm (Shanghai, China). Artificial insemination was employed for crucian carp breeding: sexually mature crucian carp were injected with domperidone maleate (Dom) at 2 mg/kg and luteinizing hormone-releasing hormone analog (A2) at 5 μg/kg one night in advance. On the following day, sperm and eggs were collected and mixed to obtain embryos, and the subsequent rearing conditions were consistent with those for zebrafish.

### 4.2. FISH

Caudal peduncles were dissected from adult zebrafish and fixed in 4% paraformaldehyde (PFA, RNase-free) for 12 h. The samples were embedded in paraffin and sectioned longitudinally at a thickness of 4 μm. DNA fragments of *scxa* and *piezo1* were amplified from zebrafish embryonic cDNA at 3 dpf using primers listed in Supplementary Table S1. Digoxigenin (DIG)-labeled RNA probes were synthesized from 1000 ng of purified PCR product using the T7 High Yield RNA Transcription Kit (Vazyme, Nanjing, China, Cat. No. TR101) and DIG RNA Labeling Mix (Roche, Basel, Switzerland, Cat. No. 11277073910). Section hybridization was performed with 3000 ng of DIG labeled probe per slide at 65 °C for 16 h. For signal detection, sections were incubated with HRP-conjugated sheep anti-DIG antibody (Roche, Basel, Switzerland, Cat. No. 11207733910) at a 1:2000 dilution, followed by tyramide signal amplification using iF488-Tyramide or iF555-Tyramide (Servicebio, Wuhan, China, Cat. No. G1222/G1223).

### 4.3. sgRNA Preparation

The CRISPR/Cas9 system was used to generate mutant fish lines. sgRNAs for each gene were designed using the online tool crisprscan.org (accessed on 8 November 2023) [27]. For zebrafish, we generated *piezo1* (ENSDARG00000076870) and *piezo2b* (ENSDARG00000112723) mutants using the conventional method of targeting each gene with a single sgRNA in the F0 generation, followed by successive crosses to obtain homozygous progeny [28]. In contrast, for crucian carp, we employed a multiplexed CRISPR strategy with three sgRNAs targeting *piezo1a* (ENSYGUG00000015181) and *piezo1b* (ENSYGUG00000047551) to enhance mutagenesis efficiency directly in the F0 generation [29].

The DNA template for sgRNA was generated by PCR using a target-specific forward primer containing the T7 promoter sequence and a universal reverse primer. The sgRNA synthesis primers are listed in Supplementary Table S1. The resulting PCR product was subsequently used for in vitro transcription using the T7 High Yield RNA Transcription Kit (Vazyme, Nanjing, China, Cat. No. TR101), and purification of sgRNAs was carried out using RNA Clean & Concentrator Kits (Zymo Research, Irvine, CA, USA, Cat. No. R1017) [30].

### 4.4. Microinjection and Genotyping

Zebrafish embryos were obtained from the natural mating of sexually mature adults (≥3 months old), whereas crucian carp embryos were acquired through artificial dry fertilization of 1-year-old sexually mature fish. Embryos at the one-cell stage were injected with 1–2 nL of mixture containing 2500 ng/μL Cas9 protein (GenScript, Nanjing, China, Cat. No. Z03389) and 600 ng/μL sgRNA.

Mutagenesis efficiency at each target site was quantified by fluorescent PCR-coupled capillary electrophoresis. Genomic DNA was extracted from whole 3 dpf embryos or adult caudal fins by alkaline lysis in 50 mM NaOH at 95 °C for 15 min, followed by neutralization with 100 mM Tris-HCl and dilution. A 1 μL aliquot of the DNA extract was used as a template for PCR amplification in a 20 μL reaction system using Green Taq Mix (Vazyme, Nanjing, China, Cat. No. P131) and genotyping primers (see Supplementary Table S1). The PCR conditions were: 95 °C for 10 min; 35 cycles of 95 °C for 30 s, 58 °C for 30 s, and 72 °C for 30 s; and a final extension at 72 °C for 10 min. The resulting amplicons were analyzed by capillary electrophoresis.

### 4.5. Establishment of Mutant Fish Lines

For the establishment of homozygous *piezo1* and *piezo2b* zebrafish lines, the injected zebrafish embryos were first reared to adulthood. Individuals with high mutation efficiency were selected as the F0 generation, which were then crossed with wild-type AB strain zebrafish to produce the F1 generation. F1 fish carrying identical mutations were screened via sequencing and subsequently subjected to inbreeding, resulting in the generation of homozygous *piezo1* and *piezo2b* mutant lines.

For crucian carp, three sgRNAs were co-injected to disrupt *piezo1* at the F0 generation. Only the injected juvenile fish exhibiting high insertion/deletion efficiency were reared to 3 mpf for subsequent phenotypic analysis.

### 4.6. Alizarin Red S Staining

Adult fish were fixed overnight in 4% PFA solution. They were then decolorized to transparency using a 5% H_2_O_2_ decolorizing solution (prepared in 1% KOH) under light exposure. Next, the fish were digested at 37 °C with a 1% trypsin solution (prepared in 1× PBST) until transparent. Staining was performed using a 0.1% Alizarin Red solution (prepared in 1% KOH) for 5 h. Finally, excess dye was removed using glycerol.

### 4.7. qRT-PCR

Total RNA was extracted from zebrafish embryos at 3 dpf using TRIzol reagent (Vazyme, Nanjing, China, Cat. No. R411), and RNA concentration was measured using a NanoDrop spectrophotometer. cDNA synthesis was conducted following the instructions provided with the HiScript III SuperMix for qPCR (+ gDNA wiper) kit (Vazyme, Nanjing, China, Cat. No. R323). The synthesized cDNA was stored at −20 °C until use.

qRT-PCR was performed using the ChamQ Universal SYBR qPCR Master Mix (Vazyme, Nanjing, China, Cat. No. Q711). Each 20 μL reaction contained approximately 50 ng cDNA and 10 pmol of each gene-specific primer. The reaction was run for a total of 40 cycles with the following parameters: initial denaturation at 95 °C for 30 s, followed by 40 cycles of denaturation at 95 °C for 10 s and annealing/extension at 60 °C for 30 s. All reactions were performed in triplicate. *eef1a1l1* was used as the internal reference gene for zebrafish. Relative gene expression was calculated using the 2^−△△Ct^ method. The qPCR primers are listed in Supplementary Table S1.

### 4.8. Data Statistical Analysis

Morphometric analysis of the alizarin red S stained IBs was performed using ImageJ 1.53t. The parameters quantified included the total number, length, and surface area of all detectable bones. All statistical analyses were carried out using GraphPad Prism 9.0. For comparison between two groups, an unpaired *t*-test with equal variance was employed. All data were displayed as mean with Standard Error of the Mean (SEM). *p* < 0.05 was regarded as statistically significant.

## 5. Conclusions

In summary, we have demonstrated that loss of *piezo1* consistently weakens IB development in both a model organism (zebrafish) and an economically important farmed species (crucian carp). This discovery serves a dual purpose: it significantly advances our fundamental understanding of skeletal biology by providing a compelling case for the role of mechanotransduction in the development of a specific bone type, and it simultaneously identifies Piezo1 as a novel and promising genetic target for the breeding of IB-reduced or IB-free aquaculture strains.

## Data Availability

The original contributions presented in this study are included in the article/Supplementary Material. Further inquiries can be directed to the corresponding authors.

## Supplementary Materials

The following supporting information can be downloaded at https://www.mdpi.com/article/10.3390/ijms262210851/s1.

## References

[1] Arratia G. (2015). Complexities of Early Teleostei and the Evolution of Particular Morphological Structures through Time. Copeia.

[2] Bao B. (2025). The Development and Evolution of Intermuscular Bones in Teleosts. JSOU.

[3] Patterson C., Johnson G.D. (1995). The Intermuscular Bones and Ligaments of Teleostean Fishes. Smithsonian Contributions to Zoology.

[4] Gemballa S., Ebmeyer L., Hagen K., Hannich T., Hoja K., Rolf M., Treiber K., Vogel F., Weitbrecht G. (2003). Evolutionary Transformations of Myoseptal Tendons in Gnathostomes. Proc. Biol. Sci..

[5] Li B., Zhang Y.-W., Liu X., Ma L., Yang J.-X. (2021). Molecular Mechanisms of Intermuscular Bone Development in Fish: A Review. Zool. Res..

[6] Cao D.-C., Kuang Y.-Y., Zheng X.-H., Tong G.-X., Li C.-T., Sun X.-W. (2015). Comparative Analysis of Intermuscular Bones in Three Strains of Common Carp. J. Appl. Ichthyol..

[7] Wan S.-M., Xiong X.-M., Tomljanović T., Chen Y.-L., Liu H., Treer T., Gao Z.-X. (2019). Identification and Mapping of SNPs Associated with Number of Intermuscular Bone in Blunt Snout Bream. Aquaculture.

[8] Xu H., Tong G., Yan T., Dong L., Yang X., Dou D., Sun Z., Liu T., Zheng X., Yang J. (2022). Transcriptomic Analysis Provides Insights to Reveal the Bmp6 Function Related to the Development of Intermuscular Bones in Zebrafish. Front. Cell Dev. Biol..

[9] Gan R.-H., Li Z., Wang Z.-W., Li X.-Y., Wang Y., Zhang X.-J., Tong J.-F., Wu Y., Xia L.-Y., Gao Z.-X. (2023). Creation of Intermuscular Bone-Free Mutants in Amphitriploid Gibel Carp by Editing Two Duplicated *Runx2b* Homeologs. Aquaculture.

[10] Kague E., Hughes S.M., Lawrence E.A., Cross S., Martin-Silverstone E., Hammond C.L., Hinits Y. (2019). Scleraxis Genes Are Required for Normal Musculoskeletal Development and for Rib Growth and Mineralization in Zebrafish. FASEB J..

[11] Kuang Y., Zheng X., Cao D., Sun Z., Tong G., Xu H., Yan T., Tang S., Chen Z., Zhang T. (2023). Generate a New Crucian Carp (Carassius Auratus) Strain without Intermuscular Bones by Knocking out Bmp6. Aquaculture.

[12] Nie C., Wan S., Chen Y., Zhu D., Wang X., Dong X., Gao Z.-X. (2021). Loss of Scleraxis Leads to Distinct Reduction of Mineralized Intermuscular Bone in Zebrafish. Aquac. Fish..

[13] Nie C.-H., Wan S.-M., Chen Y.-L., Huysseune A., Wu Y.-M., Zhou J.-J., Hilsdorf A.W.S., Wang W., Witten P.E., Lin Q. (2022). Single-Cell Transcriptomes and Runx2b−/− Mutants Reveal the Genetic Signatures of Intermuscular Bone Formation in Zebrafish. Natl. Sci. Rev..

[14] Niu S., Li X., Feng C., Zhang Z., Sha H., Luo X., Zou G., Liang H. (2025). Targeting and Editing the Second Exon of *Bmp6* Gene Results in a Silver Carp with Reduced Intramuscular Bones. Aquac. Rep..

[15] Zheng J., He C., Jiang W., Liu S., Li F., Chi M., Cheng S., Liu Y. (2023). Screening for IBs-Relative Genes by Transcriptome Analysis and Generation IBs-Less Mutants in Culter Alburnus. Comp. Biochem. Physiol. Part D Genom. Proteom..

[16] Danos N., Ward A.B. (2012). The Homology and Origins of Intermuscular Bones in Fishes: Phylogenetic or Biomechanical Determinants?. Biol. J. Linn. Soc..

[17] Meyer A. (1987). Phenotypic Plasticity and Heterochrony in *Cichlasoma managuense* (Pisces, Cichlidae) and Their Implications for Speciation in Cichlid Fishes. Evol. Int. J. Org. Evol..

[18] Tang G.H., Rabie A.B.M., Hägg U. (2004). Indian Hedgehog: A Mechanotransduction Mediator in Condylar Cartilage. J. Dent. Res..

[19] Yao W., Lv Y., Gong X., Wu J., Bao B. (2015). Different Ossification Patterns of Intermuscular Bones in Fish with Different Swimming Modes. Biol. Open.

[20] Coste B., Mathur J., Schmidt M., Earley T.J., Ranade S., Petrus M.J., Dubin A.E., Patapoutian A. (2010). Piezo1 and Piezo2 Are Essential Components of Distinct Mechanically Activated Cation Channels. Science.

[21] Li J., Hou B., Tumova S., Muraki K., Bruns A., Ludlow M.J., Sedo A., Hyman A.J., McKeown L., Young R.S. (2014). Piezo1 Integration of Vascular Architecture with Physiological Force. Nature.

[22] Sugimoto A., Miyazaki A., Kawarabayashi K., Shono M., Akazawa Y., Hasegawa T., Ueda-Yamaguchi K., Kitamura T., Yoshizaki K., Fukumoto S. (2017). Piezo Type Mechanosensitive Ion Channel Component 1 Functions as a Regulator of the Cell Fate Determination of Mesenchymal Stem Cells. Sci. Rep..

[23] Sun W., Chi S., Li Y., Ling S., Tan Y., Xu Y., Jiang F., Li J., Liu C., Zhong G. (2019). The Mechanosensitive Piezo1 Channel Is Required for Bone Formation. Elife.

[24] Wang L., You X., Lotinun S., Zhang L., Wu N., Zou W. (2020). Mechanical Sensing Protein PIEZO1 Regulates Bone Homeostasis via Osteoblast-Osteoclast Crosstalk. Nat. Commun..

[25] Zhou T., Gao B., Fan Y., Liu Y., Feng S., Cong Q., Zhang X., Zhou Y., Yadav P.S., Lin J. (2020). Piezo1/2 Mediate Mechanotransduction Essential for Bone Formation through Concerted Activation of NFAT-YAP1-ß-Catenin. Elife.

[26] Ramli, Aramaki T., Watanabe M., Kondo S. (2023). Piezo1 Mutant Zebrafish as a Model of Idiopathic Scoliosis. Front. Genet..

[27] Moreno-Mateos M.A., Vejnar C.E., Beaudoin J.-D., Fernandez J.P., Mis E.K., Khokha M.K., Giraldez A.J. (2015). CRISPRscan: Designing Highly Efficient sgRNAs for CRISPR-Cas9 Targeting in Vivo. Nat. Methods.

[28] Varshney G.K., Carrington B., Pei W., Bishop K., Chen Z., Fan C., Xu L., Jones M., LaFave M.C., Ledin J. (2016). A High-Throughput Functional Genomics Workflow Based on CRISPR/Cas9-Mediated Targeted Mutagenesis in Zebrafish. Nat. Protoc..

[29] Kroll F., Powell G.T., Ghosh M., Gestri G., Antinucci P., Hearn T.J., Tunbak H., Lim S., Dennis H.W., Fernandez J.M. (2021). A Simple and Effective F0 Knockout Method for Rapid Screening of Behaviour and Other Complex Phenotypes. Elife.

[30] Li Z., Ouyang Y., Yuan X., Zheng H., Wang J., Fan C. (2025). Essential role of RANK-NF-κB Signaling Pathway in Spinal Deformities: Insights from Largemouth Bass and Zebrafish Models. Aquaculture.

