# Supplementary material for: The Mutation of piezo1 Weakens the Intermuscular Bones in Zebrafish and Crucian Carp
Source: Int J Mol Sci. 2025 Nov 8;26(22):10851. doi: 10.3390/ijms262210851 (PMC12652723; doi:10.3390/ijms262210851)
Supplement: Supplementary file 1 [file ijms-26-10851-s001.zip › ijms-3967246-supplementary.pdf]

The mutation of *piezo1* weakens the intermuscular bones in zebrafish and crucian carp

Xinyu Zhang [1,2,†], Jinyuan Che [1,2,†], Zhuang Li [1,2], Baolong Bao [1,2,3,*] and Chunxin Fan [1,2,3,*]

[1] Key Laboratory of Exploration and Utilization of Aquatic Genetic Resources, Ministry of Education, Shanghai Ocean University, Shanghai 201306, China

[2] International Research Center for Marine Biosciences, Ministry of Science and Technology, Shanghai Ocean University, Shanghai 201306, China

[3] National Demonstration Center for Experimental Fisheries Science Education, Shanghai Ocean University, Shanghai, 201306, China.

[*] Corresponding author: cxfan@shou.edu.cn (C.F.); blbao@shou.edu.cn (B.B.) .

[†] These authors contributed equally to this work.

Supplementary Table S1

Primers used in this study for sgRNA template assembly, probe template assembly, genotyping and qPCR.

Name	*Sequence (5’ - 3’)*	Application
Universal	AAAAGCACCGACTCGGTGCCACTTTTTCAAGTTGATAACGGACT	sgRNA Template
Bottom	AGCCTTATTTTAACTTGCTATTTCTAGCTCTAAAAC	Assembly
M13-FAM	CAGGGTTTTCCCAGTCACG	Genotyping
**Zebrafish**		
Scxa	F: GAGATCCGTCAACGCAATGC<br>R: GGACACACTGGTGATGCTGA	Probe Template<br>Assembly
piezo1	F: AGTTGGCAGACATGGGACTG<br>R: CTGGGTATCACCTCGTCAGC	Probe Template<br>Assembly
piezo1	TAATACGACTCACTATAGCTGCTTGCAGTACGTGTGTTTTAGAGC<br>TAGAA	sgRNA Template<br>Assembly
piezo2b	TAATACGACTCACTATACGTTCTCGATGGGCACGGGTTTTAGAGC<br>TAGAA	sgRNA Template<br>Assembly
piezo1	F: TGTAAAACGACGGCCAGTGGATGCTGAGGACACGTCAA<br>R: GTGTCTTCTCACCAGGGCACCCAAG	Genotyping
piezo2b	F: TGTAAAACGACGGCCAGTTGCCCATAATTCCCGTTTGTG<br>R: GTGTCTTAGGCATTTGAGACTTCGCGG	Genotyping
eef1a1l1	F: ACCTACCCTCCTCTTGGTCG<br>R: GGAACGGTGTGATTGAGGGAA	Reference for qPCR
piezo1	F: GAGAGGATGCGGCTTCTCAA<br>R: CCACATGGTGAATCCGTCCA	qPCR
piezo2b	F: ACCAGCCAGTTTGAGGATGG<br>R: ACCACTGACTTTAGCATAGCTGTA	qPCR
**Crucian Carp**		
piezo1a-T1	TAATACGACTCACTATAGGCATTATCAAGGCTGGGGAGTTTTAG<br>AGCTAGAA	sgRNA Template<br>Assembly
piezo1a-T2	TAATACGACTCACTATAGGTGTGCCGTGGCACGCAGGGTTTTAG<br>AGCTAGAA	sgRNA Template<br>Assembly
piezo1a-T3	TAATACGACTCACTATAGGGTGAGGCTGGAGAGGAAGGTTTTAG<br>AGCTAGAA	sgRNA Template<br>Assembly
piezo1a-T1	F: TGTAAAACGACGGCCAGTCTCCATTCCACAGGACACAC<br>R: GTGTCTTGCCTGGACCAACTACTAACC	Genotyping
piezo1a-T2	F: TGTAAAACGACGGCCAGTTGTAGAAGGAGAAGATGAGGGAA<br>R: GTGTCTTGTCCAACTATTATGCTAAGCCTG	Genotyping
piezo1a-T3	F: TGTAAAACGACGGCCAGTACAGCTCTGTCTCCTTGAAC<br>R: GTGTCTTCAAATTGGGGGCTTTGTCCA	Genotyping

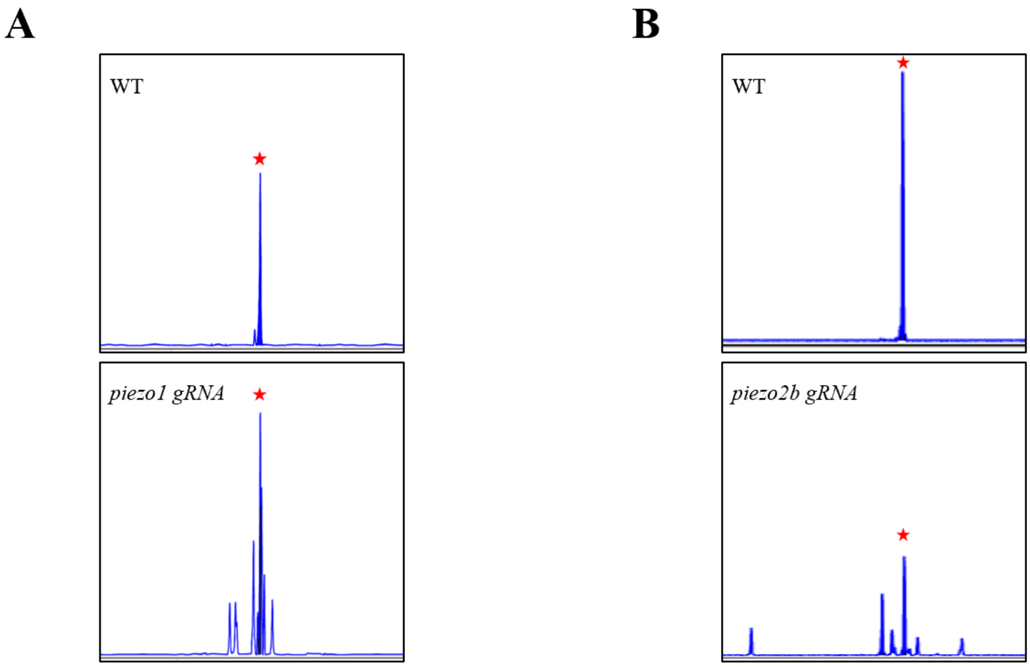

Supplementary Figure S1. Analysis of *piezo1* (A) and *piezo2b* (B) targeting efficiency by fluorescent PCR-coupled capillary electrophoresis. The x-axes (size) and y-axes (signal intensity) of the peak diagrams for WT and mutant fish are aligned. Red stars indicate the position of the WT peak.
